# Drug resistance and Cancer stem cells

**DOI:** 10.1186/s12964-020-00627-5

**Published:** 2021-02-15

**Authors:** Yuan Li, Zhenning Wang, Jaffer A. Ajani, Shumei Song

**Affiliations:** 1 Department of Gastrointestinal Medical Oncology, The University of Texas MD Anderson Cancer Center, 1515 Holcombe Boulevard, Houston, TX 77030-4009 USA; 2 Department of Surgical Oncology and General Surgery, First Hospital of China Medical University, Shenyang, 110001 People’s Republic of China

**Keywords:** Drug resistance, Cancer stem cells, EMT and TME

## Abstract

Therapy resistance is a major problem when treating cancer patients as cancer cells develop mechanisms that counteract the effect of therapeutic compounds, leading to fit and more aggressive clones that contribute to poor prognosis. Therapy resistance can be both intrinsic and/or acquired. These are multifactorial events, and some are related to factors including adaptations in cancer stem cells (CSCs), epithelial-mesenchymal transition (EMT), deregulation of key signaling pathways, drug efflux through ABC transporters, acquired mutations, evading apoptosis, and activation of DNA damage response among others. Among these factors, CSCs represent the major source of therapy resistance. CSCs are a subset of tumor cells that are capable of self-renewal and multilineage progenitor expansion that are known to be intrinsically resistant to anticancer treatments. Multiple clones of CSCs pre-exist, and some can adopt and expand easily to changes in the tumor microenvironment (TME) and/or in response to radio- and chemotherapy. A combination of both intrinsic and extrinsic factors contributes to CSC-mediated therapy resistance. In this review, we will focus on CSCs and therapy resistance as well as suggest strategies to eliminate CSCs and, therefore, overcome resistance.

Video abstract.

**Supplementary Information:**

The online version contains supplementary material available at 10.1186/s12964-020-00627-5.

## Introduction

Tumors harbor heterogeneous clones of cancer stem cells (CSCs) representing the fundamental properties for survival [1]. In recent years, addressing the origin of intratumor heterogeneity (ITH) has become one of the core challenges for overcoming therapy resistance. The clonal evolution model considers tumors to be the result of random evolution, while the CSC model considers tumor heterogeneity due to the presence of CSCs [2, 3]. CSCs are defined as “cells that have the ability to self-renew and also create a progeny”. Therefore, the bulk of cancer mass is formed by the differentiated and expanded progeny with high proliferation potential while harboring small populations of various CSCs. At present, cure of tumor remains a major challenge for oncologists due to advanced stage of most cancers and the presence of resistant CSC clones [4, 5].

It has been proposed that targeting CSC subpopulations can result in tumor elimination thus no likelihood of tumor relapse [6]. CSCs evade conventional therapies by remaining dormant, have increased DNA repair capacity, turn off apoptotic pathways, manage reactive oxygen species (ROS) and reactive nitrogen species (RNS) in a highly competent manner, and by manipulating TME (tumor microenvironments) to their advantage [7].

## Potential CSCs markers and therapy resistance

In essence, CSCs are defined by their intrinsic ability to propagate the tumor, thereby explaining the alternative names “tumor initiating cells” or “tumorigenic cells” [8]. CSCs can be identified by various surface markers. To date, several CSC markers in different tumor types have been proposed and validated through cell lines, patient samples, and xenotransplantation of CSCs in animal models. Followings are the CSC biomarkers of interest:

*CD133*, also known as prominin-1, a glycoprotein with five transmembrane domains, was identified from mouse neuro-epithelial stem cells and human hematopoietic stem cells. The expression of CD133 is not restricted to normal stem cell, but also found in many tumor types. Dirks et al. discovered that CD133 expressed in brain tumors and used CD133 as a CSC marker to identify brain CSCs [9]. CD133 was also identified in various other tumors including breast, stomach, colon, prostate, liver, pancreatic, ovarian, lung cancer, and head and neck squamous cell cancers [10–14]. CD133-expressing CSCs exhibited self-renewal potential and over-expression of CD133 has been associated with poor prognosis and reduced overall survival in gastric adenocarcinoma and several other tumor types [15].

High expression of CD133 is associated with drug resistance. The presence of CD133-positive CSCs in lung cancer increases the expression of the ABC transporter ABCG2, resulting in lung cancer resistance to first-line drugs such as platinum and paclitaxel. Studies have shown that low-dose platinum therapy can cause DNA damage rather than cell death, which can induce ABCG2 upregulation and further increase the number of CD133-positive cells. Specific ABCG2 inhibitor Pantoprazole or ABC transporter inhibitor Verapamil can reduce tumor resistance to platinum [16]. It has been reported that CD133 mediates cisplatin resistance that can overcome by inhibition of CD133 [17].

*CD44*, is a transmembrane receptor for hyaluronic acid (HA) and many other extra-cellular matrix (ECM) components and a coreceptor for growth factors and cytokines. It is reported that CD44 is associated with increased potential for tumor initiation and progression [18]. CD44 is an important cell surface molecule that can sense, integrate, and transduce cellular microenvironment signals to membrane-associated cytoskeleton proteins or to nuclei, thereby regulating the expression of various genes that control cell behaviors. Increasing evidence suggests that CD44, especially the CD44v subtype, is a CSC biomarker and a key regulator of cancer stemness, metastases, and response to therapy [19].

*ALDH1,* is a member of the aldehyde dehydrogenase (ALDH) superfamily of enzymes, which comprises 19 human isozymes. ALDH1 is known to participate in many important physiological biosynthetic pathways, and certain ALDH1 activities have been shown to be crucial in the detoxification of specific endogenous and exogenous aldehyde [20]. Many studies indicate that ALDH1 is involved in therapy resistance. ALDH1 controls the oxidation of aldehydes to corresponding acids, and ALDH-mediated detoxification of toxic aldehyde intermediates produced in cancer cells treated with certain therapeutic agents has been proposed to confer therapy resistance to ALDH1+ tumor cells [21]. In esophageal cancer, ALDH1^high^ is associated with reduced response in chemoradiation and chemotherapy [22]. Meng’s study indicates that ALDH1 expression is also correlated to the platinum resistance in ovarian cancer by regulating cell cycle checkpoints and the DNA repair pathway [23]. Using ALDH1 sorting, Nguyen et al. found that ALDH1+ CSCs had higher tumorigenicity in mice and were more resistant to therapy than ALDH1− gastric cancer cells [24]. Study from our laboratory also demonstrated that ALDH1 expression levels predict response or resistance to preoperative chemoradiation in resectable esophageal cancer patients and we found that sorted ALDH1+ cells were more resistant and had an aggressive phenotype than ALDH1- cells [22]. Targeted inhibition of ALDH1 could prevent recurrence of the tumor driven by ALDH1+ CSCs [25]. Therefore, as a detoxifying enzyme, ALDH1 is highly expressed in CSCs to alleviate the toxic effects of ROS and to control the cell cycle so that the cells have enough time for DNA repair, which enables CSCs to resist therapy.

*CD166*, also named as activated leukocyte cell adhesion molecule (ALCAM), is a glycoprotein belonging to the immunoglobulin superfamily of adhesion molecules [26]. Unlike other adhesion molecules such as E-cadherin which are usually down-regulated during malignant transformation, CD166 often shows increased expression in certain cancers [27]. Overexpression of CD166 in papillary thyroid carcinoma (PTC) was independently associated with a shorter progression-free survival, higher nodal and tumor stages suggesting that CD166 may be a potential therapeutic target to treat PTC [28]. CD166 has been implicated as CSCs marker in many cancer types such as colon, stomach and head/neck [24, 29]. Recent study from Satar NA et al. demonstrated that CD166/EpCAM/CD44 triple positive clones mediated therapy resistance and putative CSC characteristics in human non-small cell lung cancer cells [30].

*CD49f,* (integrin subunit alpha 6, ITGA6), is also identified to be a CSC surface marker, and found to correlate with tumor spheres formation capacity and in vivo self-renewal ability in lung cancer [31]. In glioblastoma CSCs (GCSCs), CD49f has also been proposed to be an important regulator of stemness [32]. It has been reported that CD49f is associated with radiation therapy resistance and CD49f + population-mediated taxane resistance [33, 34].

*CD24*, also known as heat-stable antigen in mouse, is significantly upregulated in different cancers compared to their benign counterparts. In many human cancers, CD24 overexpression is highly associated with adverse prognostic features such as lymph node metastases, advanced clinical stage and shorter overall survival [35]. It is identified to be a CSC marker in bladder cancer [36], hepatocellular carcinoma [37]. In Terence Kin Wah Lee’ s study, CD24+ cells were more quiescent, with a greater ability to form tumors in Non-obese diabetic (NOD) /Severe combined immune deficient (SCID) mice, and an ability to self-renew, differentiate, and metastasize, especially upregulated in residual resistant tumors upon cisplatin treatment when compared with untreated tumors [37].

*CD9*, motility-related protein-1, is involved in cell fusion, adhesion, motility, proliferation, metastases and signaling [38, 39]. Some studies have reported that CSCs can contribute towards tumor formation by upregulating CD9, thereby maintaining the tumor cell population [40, 41]. In human B-acute lymphoblastic leukemia, CD9 plays important roles in attributing CSC properties, and the CD9+ cells exhibited therapy resistance [42].

## Deregulated developmental signaling that govern CSCs and therapy resistance

**Fig. 1:**
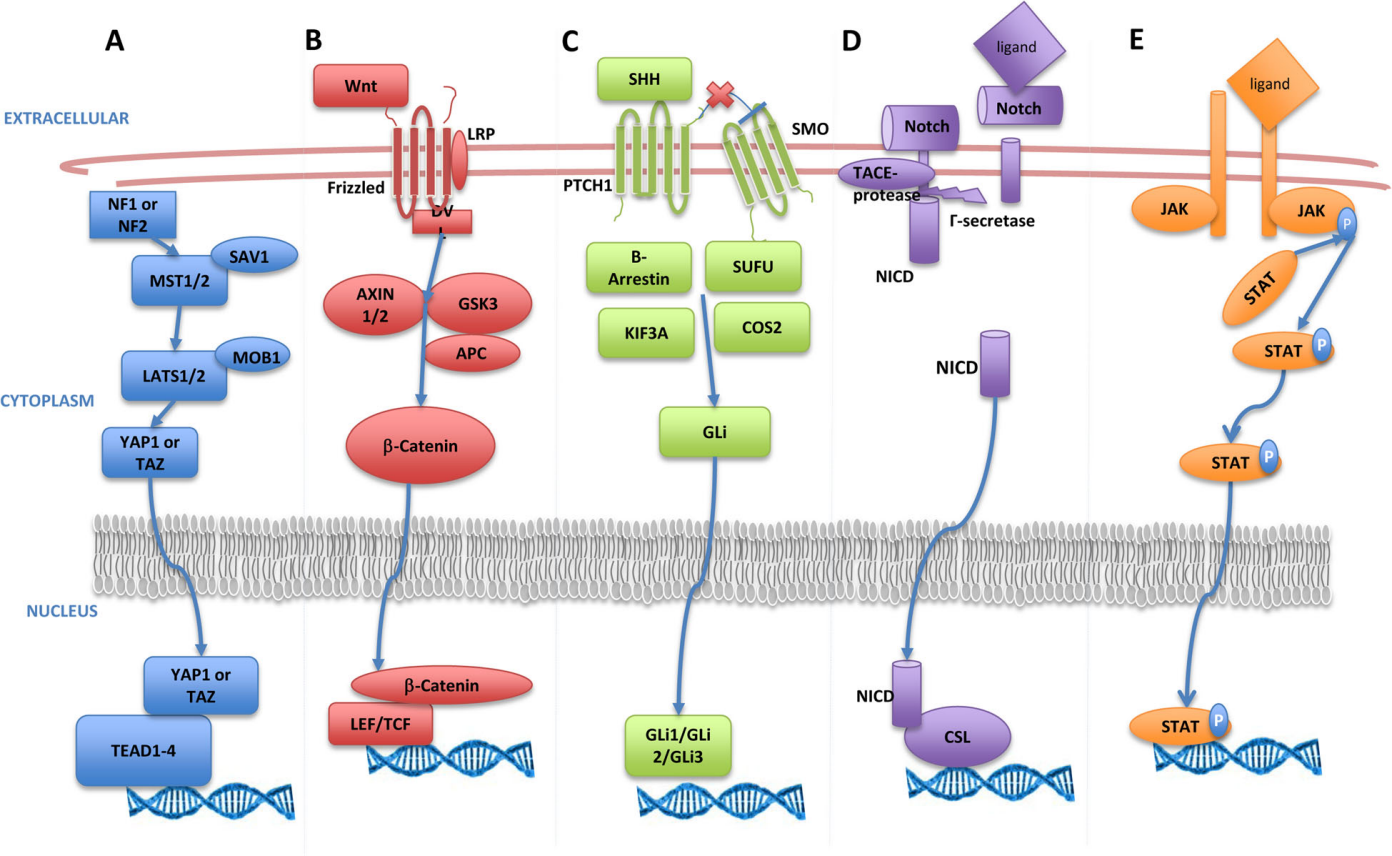
Developmental signaling that govern CSCs and therapy resistance. **a.** Hippo signlaing consists of a core kinase cascade in which Mst1/2 forms a complex with the adaptor SAV1 that phosphorylates the kinases LATS1/2. LATS1/2 then phosphorylates and represses the transcriptional coactivators YAP1 and TAZ by promoting their degradation and cytoplasmic retention. When deregulation of the Hippo signaling by deletion or mutation of these kinases, YAP/TAZ accumulate in the nuclear and binds to its transcription factor Tead1–4 and upregulation of its target genes involving cell proliferation, CSCs properties and drug resistance. **b.** When SHH reaches its target cell, it binds to the PTCH1 receptor. The binding of SHH relieves SMO inhibition, leading to activation of the GLI transcription factors. Activated GLI accumulates in the nucleus and controls the transcription of hedgehog target genes. **c.** The Wnt/β-catenin pathway is important for CSC maintenance. In the canonical pathway, Wnt ligands bind to transmembrane Frizzled receptor, leading to the recruitment of Dvl protein. Dvl triggers the cytoplasmic accumulation of β-catenin which translocates into the nucleus, where it forms a complex with TCF/LEF that control genes in CSCs and therapy resistance. **d.** Notch signaling is activated through cell-to-cell contact. Ligands bind to Notch receptors on the target cell. This allows intracellular cleavage through γ-secretase, producing NICD. NICD translocates to the nucleus binding with CSL complex, triggering transcription and leading to CSC maintenance, metastasis and chemo-resistance. **e.** JAK-STAT signaling is made of three major proteins: cell-surface receptors, JAKs, and STATs. Once a ligand binds to the receptor, JAKs phosphorylates the receptor (gp130) which allows STAT proteins binds and be phosphorylated by JAKS to form a dimer. The phosphorylated STAT dimer enters the nucleus, binds to DNA, and causes transcription of target genes

*Hippo*/*YAP1* *pathway* is a highly conserved signaling pathway that regulates cell fate, apoptosis, proliferation and stem cell maintenance in various species [43, 44]. The Hippo pathway components, including a major kinase cascade and scaffold proteins, have been established in both Drosophila and mammals [45]. In mammals, the Hippo pathway consists of a core kinase cascade in which Mst1/2 form a complex with an adaptor protein Sav1 that phosphorylates kinases Lats1/2. Lats1/2 then phosphorylates and represses the transcriptional coactivators YAP1 and TAZ by promoting ubiquitination, degradation, and cytoplasmic retention (Fig. 1a).

Recent emerging data suggest that YAP1 regulate CSCs properties and confer therapy resistance [46, 47]. Overexpression of YAP1 and its activation (nuclear localization) are associated with poor prognosis in several tumor types, including gastric adenocarcinoma (GAC). We have demonstrated that YAP1 is overexpressed in gastroesophageal cancer and mediates CSC properties through its target SOX9 [48]. Studies from our laboratory also found that YAP1 strongly mediates chemo- and radiation resistance through upregulation EGFR and CDK6 in esophageal cancer respectively [49, 50]. YAP1 has been reported as conferring resistance to cisplatin in human oral squamous cell carcinoma [51]. Others surmised that YAP1 is often the terminal node of many oncogenic pathways [52] and a signaling hub of the tumor microenviroment [53].

In addition, YAP1 was reported to be responsible for target therapy resistance. Lin L et al. reported that YAP1 enhances resistance to RAF and MEK-targeted cancer therapies resistance, revealing that YAP1 and RAF or MEK combined inhibition of synthetic lethality is a promising strategy to enhance treatment response and patient survival [47]; while Lee JE et al. found that YAP1 inhibition restores the sensitivity of EGFR-TKI in lung adenocarcinoma having primary or acquired EGFR-TKI resistance [54].

It has been reported that another hippo downstream effector, TAZ, promotes the self-renewal and tumor-seeding potentials of CSCs and confers CSC-like properties on differentiated non-CSC cells in different cancer contexts [55]. Recent evidence suggests that TAZ is also associated with therapy resistance. Zhan z et al. reported that TAZ, is associated with drug resistance of pancreatic cancer [56], high expression of TAZ in lung adenocarcinoma cells and cell lines is associated with cisplatin resistance. TAZ inhibition restores the sensitivity of cisplatin through AKT/mTOR signaling in lung adenocarcinoma [57].

*Wnt/β-catenin pathway* is an ancient and evolutionarily conserved pathway which regulates embryonic development [57]. The canonical Wnt/β-catenin signaling cascades are involved in self-renewal of stem cells and proliferation or differentiation of progenitor cells, whereas non-canonical Wnt signaling cascades are involved in maintenance of stem cells, and directional cell movement (Fig. 1b). Both canonical and non-canonical Wnt signaling cascades play key roles in the development and evolution of CSCs [58].

The abnormal activation of Wnt signaling has implicated in many cancers including hepatoblastoma, colorectal cancer, multiple myeloma, and GAC [59]. It has been shown that factors secreted by fibroblasts, such as hepatocyte growth factor (HGF), activate the Wnt/β-catenin pathway and CSC expansion in vivo and in vitro. In a colon cancer model, CSCs with high Wnt signaling activity appear to be adjacent to stromal myofibroblasts, which secrete multiple factors to maintain the active Wnt/β-catenin pathway, thereby ensuring the stemness characteristics of its neighboring cells [60]. In malignant pleural mesothelioma, Wnt/GSK3β/β-catenin pathway is found to be upregulated and Wnt-driven autocrine production of IL-8 and IL-1β contributes to upregulate ABCB5 which is predictive of poor response to chemotherapy [61]. Emons G et al. report that chemoradiotherapy resistance in colorectal cancer cells is mediated by Wnt/β-catenin signaling [62].

*Hedgehog pathway (Hh)* is critical during embryonic development. It is involved in the patterning of the neural tube, lung, skin, axial skeleton, and gastrointestinal tract [63]. Binding of Hh ligands, relieves the inhibitory effect of their Patched (PTCH) transmembrane receptors on Smoothened (SMO), which is also located in the cell membrane. Subsequently, the signaling cascade initiated by SMO leads to activation and nuclear localization of GLI transcription factors, which drive expression of Hh target genes; most of the target genes are involved in proliferation, survival, and angiogenesis (Fig. 1c) [64].

Hh signaling has been associated with cell fate determination, self-renewal [65] and CSC regulation in many cancer types including leukemias [66], multiple myeloma [67], gliomas [68], breast cancer [69], pancreatic cancer [70], prostate cancer [71], lung cancer [72], melanoma [73], and gastrointestinal cancers [74, 75]. It has been reported that activation of Hh signaling contributes to therapy resistance and Hh signaling contributes to tumor regrowth after chemoradiotherapy and a target to improve radiation response [76]. Kobune M found that drug resistance is dramatically restored by Hh inhibitors in CD34+ leukemic cells [77]. Hh signaling affects the sensitivity of hepatoma cells to therapy through the ABCC1 transporter [78].

*The Notch pathway,* has been extensively explored as a CSC-realted pathways in multiple tumor types. The pathway is activated upon ligand binding to the Notch receptor, which is subsequently cleaved by the ADAM family proteases and γ-secretase to release the Notch intracellular domain (NICD). NICD translocates to the nucleus, binds to transcription factor CSL, and converts the complex from a repressor to an activator of Notch genes (Fig. 1d). Notch activation has been proposed as vital to CSC populations for maintaining stemness, enhancing therapy resistance, and promoting a hypoxic niche [79]. Notch signaling pathway plays an important role in normal stem cells proliferation, differentiation, and apoptosis. It is also reported that Notch signaling is crucial for cell survival and self-renewal properties of CSCs [57, 80].

Notch signaling is one of the most important cascades involved in therapy resistance in tumor cells [71]. Zhang Y et al. reported that Notch signaling regulates self-renewal and platinum resistance of CSCs in human non-small cell lung cancer [31]. Another report suggested that Notch signaling induces platinum resistance in a HES1-independent manner. Furthermore activation of the Notch signaling pathway is involved in osteosarcoma resistance [81]. Silencing of Notch1 suppressed AKT pathway, reduced EMT, and enhanced the sensitivity of TNBC cells to cisplatin and doxorubicin [82].

*JAK/STAT pathway*, plays a critical role in various cytokines and growth factors signaling that affect various cellular functions, such as proliferation, growth, and immune response. JAK/STAT signaling is reported to be involved in maintaining embryonic stem cell self-renewal properties, hematopoiesis, and neurogenesis [83]. It is also reported that inhibiting JAK/STAT pathway can block CSC self-renewal [84]. In the breast cancer, expression of a variety of lipid metabolic genes, including carnitine palmitoyltransferase 1B (CPT1B), which encodes the key enzyme for fatty acid b-oxidation (FAO) is also blocked by the inhibition of JAK/STAT pathway. Human breast-cancer-derived data suggest that the STAT3-CPT1B-FAO pathway promotes cancer cell stemness and therapy resistance. In addition, cells when treated with FDA-approved JAK inhibitor Tofacitinib, became sensitive and underwent apoptosis when combined with doxorubicin [85].

## Mechanisms of CSC-mediated therapy resistance

CSC-mediated therapy resistance appears to be associated with their dormancy/slow-cycling, and/or expression of efflux transporters, avoidance of apoptosis and Non-coding RNAs mediated drug resistance etc.

### CSC dormancy, plasticity and drug resistance

Tumor dormancy, a clinically undetectable state of cancer, contributes the development of multidrug resistance (MDR), minimum residual disease (MRD), tumor outgrowth, cancer relapse, and metastases. CSCs can mediate therapy resistance through dormancy. Cellular dormancy means that cells are recruited into the G0-phase of the cell cycle but remain capable of cell division in response to mitotic stimulation [85]. Chemotherapy and irradiation are mainly effective against proliferating cells. It is likely that dormant tumor cells comprise both CSC and non-CSC populations [86]. Study from the Massague J laboratory demonstrated that latency competent cancer (LCC) cells show stem-cell-like characteristics and express SOX2 and SOX9 transcription factors, which are essential for their survival and resistance to therapy in host organs under immune surveillance and for metastatic outgrowth under permissive conditions [87].

### ATP-binding cassette transporter (ABC transporter)

ABC transporters are proteins that allow transmembrane transportation of different substrates using the energy produced by ATP hydrolysis. These proteins are generally located on the membrane of the cell, which can protect cells from harmful toxins [88]. ABC transporters contain ABCG2, ABCB1, and ABCC1, etc. Among them, ABCG2 has the ability to transport drugs such as doxorubicin and methotrexate. ABCB1, known as P-glycoprotein, can be expressed in more than half of chemo-resistant tumors [89]. Recent study indicates that downregulating ABCG2 can enhance the chemo-sensitivity of breast CSCs [90]. Similarly, SUN et al. [91] also found that CSCs showed enhanced chemo-sensitivity when siRNAs that blocked ABC transporter expression were added to breast CSC culture media along with drugs. Surprisingly, ABC transporter proteins such as multi-drug resistance protein-1, leading to therapy-resistance of CSCs is controlled by Hh signaling [92]. Therefore, ABC transporter proteins can be used as a surface markers for CSC identification, and their ability to transport drugs itself enables CSCs to prosper.

### Avoidance of apoptosis through rho family

Rho protein, a member of small GTPases, is highly conserved and plays an important role in pathological processes including cancer progression, inflammation and wound repair [93]. Rho-associated protein kinase (ROCK), the effector of Rho, is also proved crucial in cancer progression. Recent study found that the Sox2 gene can regulate the motility of colorectal cancer cells and promote tumor progression through the Rho-ROCK signaling pathway [94]. In the study of targeting of Rho-ROCK pathway in melanoma and breast CSCs, CSC motility and invasiveness decreased [95]. Similarly, small molecule inhibitors targeting ROCK could inhibit the expression of survivin by blocking the Rho pathway and increase the sensitivity of pancreatic CSCs to drugs [96]. In conclusion, activation of the Rho-ROCK pathway promotes survivin expression, and survivin acts as an anti-apoptotic protein to protect CSCs from therapy-induced apoptosis, thus enabling CSCs to resist therapy and strengthen stemness.

### Non-coding RNAs (nc-RNAs), CSCs and therapy resistance

Recent studies have shown that different types of nc-RNAs, such as microRNAs (miRNAs) and long-chain non-coding RNAs (IncRNAs) can control growth and division of CSCs and disease progression by regulating transcription factors and downstream signaling pathways [97–100] Therefore, the effects of non-coding RNAs on intracellular signaling pathways and cell stemness maintenance are the basis for many diseases including tumors [100, 101]. Accumulated evidence confirms that miRNAs are critical for the maintenance, self-renew, and differentiation of CSCs [102]. For example, study by Liu et al. [103] demonstrated that miR-125b upregulated by the Snail via Wnt pathway can promote CSC hematopoiesis and therapy resistance. In the study of pancreatic cancer, it was reported that simultaneous inhibition of miR-21 and miR-221 can reduce the number of CSCs and reduce differentiation, leading to a decline in the overall proliferative, invasive ability and therapy resistance [104]. While Inc-RNA is a group of nc-RNAs longer than 200 nucleotides, studies have shown that Inc-RNA is closely related to CSCs. Studies in lung adenocarcinoma have shown that IncRNA-ROR regulates the expression of EMT-associated with tumor invasion, metastases, and therapy resistance [105]. Wang et al [106] reported that in prostate cancer, IncRNA HOTAIR can be induced by gemcitabine, and can promote the self-renewal and migration ability of CSCs. In summary, miRNAs and LncRNAs mainly regulate CSCs by controlling the expression of intracellular proteins and the activation of related signaling pathways, which enables CSCs to maintain their stemness and therapy resistance.

### Other stemness genes that mediate therapy resistance

#### Numb protein

Numb protein, involves cell development, adhesion and migration, cell misalignment, and endotoxin, and ubiquitination of the target protein [107]. In prostate cancer, low or negative Numb CSCs preferentially express Notch and Hh downstream and stem cell–associated genes, enrich a castration-resistant prostate cancer cell subpopulation [107]. Phosphorylation of Numb by NANOG destabilizes Numb-p53 complex, leading to p53 proteolysis, then promotes self-renew and tumorigenesis in liver cancer [108].

#### Musashi (MSI)

Musashi, underwent a duplication event in vertebrates giving rise to two homologs: Musashi1 (MSI1) and Musashi2 (MSI2). As a member of RNA binding protein family, it is capable of maintaining the infinite proliferation of stem cells through transcriptional regulation or activation of related protein expression [109]. By down-regulating pro-apoptotic genes, overexpression of MSI1 in glioblastoma can protect tumor cells from apoptosis induced by drugs [110]. The study found that MSI1 as a stem gene in colorectal cancer cells is a key regulator of CD44^+^ CSC development and enhances tumor stem cell therapy resistance by triggering the formation of anti-apoptotic stress granules (SGs) [111]. FANG’s study showed that MSI2 can up-regulate the expression level of the self-renewing gene Lin28A in hepatocellular CSCs to achieve CSC therapy resistance, and knock down of MSI2 gene leads to changes in CSC self-renewal and therapy resistance [112]. Therefore, MSI proteins play an important role in the anti-apoptotic process of CSCs that might be the molecular basis for CSCs to resist drugs.

#### Bmi1

The chromatin modifier Bmi1 is required for self-renewal of hematopoietic stem cells as well as for self-renewal of neural, mammary-gland, and prostate gland stem cells [113]. Bmi 1 functions by modifying histones and repressing genes that regulate apoptosis (P19 and p53) and senescence (p16) in stem cells but not in their differentiated progeny [114]. It has been reported that Bmi1 is responsible for the resistance to the tyrosine kinase inhibitors (TKIs) in a *BCR-ABL1*-independent way and co-expressed CD26+ in leukemic stem cells of chronic myeloid leukemia [115]. Study from Tang D’s group demonstrated that Bmi1 confers resistance to tamoxifen in estrogen receptor positive breast cancer [116].

#### Toll-like receptors 4 (TLR4)

TLR4 is a transmembrane protein, its activation leads to initiation of intracellular NF-kB signaling pathway and production of inflammatory cytokines associated with the innate immune system [117]. While NF-κB plays an important role in the regulation of immune response, and its dysregulation is considered to be related to tumorigenesis. In gliomas, the interaction of lipopolysaccharide (LPS) with TLR4 can induce tumor stem cell proliferation, and therapy resistance. At the same time, the cytotoxic T cell killing ability can also be alleviated by LPS [118]. In human hepatocellular carcinoma, it reported that the expression of TLR4 was associated with stemness of CSCs and TLR4 promoted tumor invasion, metastases, and might serve as a surface marker for CSCs [119]. Therefore, activation of TLRs and its down-stream signal pathways (NF-κB, etc.) enhance the stemness of CSCs and increase the expression level of cytokines (TNF-α, IL-6, etc.) that are associated with CSC therapy-resistance.

## CSCs niche, TME and drug resistance

The tumor microenvironment (TME) consists of stromal cells, immune cells, cytokine networks, chemokines and growth factors, hypoxic regions and ECM. TME stimulates CSC self-renewal, angiogenesis and remodeling immunity, providing other environments that are conducive to CSC tumor invasion and metastasis and dynamic changes [120]. CSCs niche modulates the Wnt/β-catenin, Notch, and Hh signaling pathways and/or interrupts the master transcriptional regulators like NANOG, OCT-4, and SOX-2 etc. to maintain the stemness of CSCs. Under specific microenvironmental stimuli, certain cancer cells exhibit plasticity, which enables them to resume proliferation through EMT [121]. For example, Chang et al [122] revealed that the activation of the Hh signaling pathway in the TME can transform common prostate cells into stem cells. The TME mainly affects the process of tumor progression and evolution, and the CSCs niche that exists in the TME plays an important role in the origin and evolution of the tumor, which proves that the TME has an important influence on the development stages of tumors.

To date, many therapy resistance mechanisms involving the TME and CSCs niche have been identified across cancer types and these mechanisms have been classified into a range of categories including physical barriers to treatment and cell-adhesion-associated drug resistance [123].

## Strategies to target CSCs and overcome therapy resistance

Because of their role in drug resistance and tumor metastasis, CSCs have contributed significantly to adverse outcomes of patients. In terms of reducing CSCs to improve the prognosis of patients, therapies that target key molecules for CSC maintenance seem theoretically feasible. Several new therapies targeting stem-associated genes and pathways have been proposed to specifically eradicating CSCs [124].

### Target deregulated CSCs signaling

As mentioned earlier, dysregulation of developmental signaling pathways has been shown to be associated with the oncogenic function of CSCs. Targeting pathways that lead the normal stem cells to CSCs open a new dimension for treating cancers associated with the high rate of recurrence and therapy resistance. Several novel strategies targeting CSC affecting pathways alone and in combination with different therapeutic agents are in clinical trials. For example, EMT and CSC marker expression were significantly enhanced in resistant ovarian cancer cells, which was accompanied by activation of PI3K/Akt/mTOR signaling. Compared with single cisplatin treatment, combined treatment with pathway inhibitor and cisplatin significantly disrupted the colony formation ability, induced higher ROS levels and more apoptosis in resistant ovarian cancer cells. Furthermore, the combination approach effectively inhibited the PI3K/Akt/mTOR signaling pathway, reversed EMT, and decreased CSC marker expression [125]. In liver cancer, the use of curcumin (NF-kB signaling pathway inhibitor) to block NF-kB can specifically target the CSC population, and suggests the potential for the combined inhibition of NF-kB and HDAC signals can be used to treat patients with poor prognosis [126].

In osteosarcoma, tankyrase inhibitor (IWR-1) inhibited tumor progression associated with specific down-regulation of TCF/LEF transcriptional activity and nuclear β-catenin expression, suggesting that targeting the Wnt/β-catenin pathway can eliminate the CSC population. In cervical cancer cell line, curcumin inhibited proliferation, invasion, stemness of cervical cancer cells through impairing Wnt/β-catenin and NF-Kβ pathways [127] The combination of conventional chemotherapy with Wnt/β-catenin inhibition can improve therapeutic effect by eliminating aggressive osteosarcoma CSCs and reducing therapy resistance [128]. Many Wnt/β-catenin signaling inhibitors have been developed and in the preclinical and clinical trials such as PKF115–584 (CGP049090) has been tested and shown inhibiting the growth of HCC cells in xenografts [129].

The importance of Hippo/YAP1 and deregulation of the Hippo pathway during cancer development and progression are emerging [130]. Our recent report demonstrated that, CA3, a novel potent YAP1 small molecule inhibitor effectively suppressed CSC properties and reduced the fraction of ALDH1+ cells enriched in radiation resistant cells [131]. Moreover, CA3 and 5-FU synergistically inhibited EAC growth, especially that of high YAP1-expressing and resistant cells [131]. YAP1 antisense oligo has been tested in the preclinical and clinical trials through collaboration between Ionis and MDACC (unpublished data).

Recently, FDA approved three new drugs that can target CSCs. Vismodegib is a hedgehog inhibitor that targets a subset of CSCs in basal-cell carcinoma [132]. Vismodegib has also been tested in preclinical models and clinical trials in other solid tumors, such as esophageal cancer. The BCL-2 inhibitor venetoclax selectively killed AML stem cells, and demonstrated that 60% of patients receiving venetoclax (with other agents) had completely clinical response [133]. Similarly, we also found AT101, another pan BCL-2 inhibitor target CSC genes-YAP1/SOX9 and proved effective in esophageal and gastric cancer patients in the preclinical and clinical setting (manuscript in GUT, 2021 in press).

### Target CSCs markers

According to characteristics of CSCs, integrin αvβ3 (integrin αvβ3) acts as a cell surface adhesion molecule that can induce stemness [134]. In breast cancer, lung cancer, and pancreatic cancer, stemness is induced by KRas/RalB/NF-κB, which is expected to be an effective target for inhibiting stemness [135]. In nasopharyngeal carcinoma, FoxM1 is significantly associated with stem cell-related clinicopathological features, including advanced clinical stages, tumor recurrence, and distant metastases. At the same time, FoxM1 is closely related to the expression levels of stem cell markers such as Nanog, Sox2 and OCT4 in tumor samples, and promoted the expression of these stem-related genes in vitro. In addition, FoxM1 gives cancer cells self-renewal properties by increasing the side population (SP) cells and forms larger and more numerous tumor spheres [136]. Song et al. [137] found that the mitochondrial membrane of CSCs has a high PRX3 gene expression in colon cancer cells. FoxM1 can stimulate overexpression of CD133 and PRX3, to regulate the stemness of colon cancer stem cells. In addition, targeting glioma stem cells through combined BMI1 and EZH2 inhibition proved more effective than either agent alone both in culture and in vivo, suggesting that strategies that simultaneously target multiple epigenetic regulators within glioblastomas may be effective in overcoming therapy resistance caused by ITH [138].

### Targeting miRNA/LncRNAs that associated with CSCs

Recently, lncRNA and miRNA have been discovered as new targets for affecting stemness. In triple-negative breast cancer (TNBC), LNC00284, also known as LNCRNA-NRAD1, is associated with worth patient outcomes. Targeting NRAD1 in TNBC tumors using antisense oligonucleotides reduced cell survival, tumor growth, and the number of cells with CSC characteristics [139]. In cholangiocarcinoma (CCA) lnc-PKD2–2-3 increased CD44, CD133 and OCT4 expression as well as the CD44 + CD133+ cell proportion, raised tumor sphere forming efficiency and enhanced tharapy resistance to 5-FU in TFK-1 and Huh-28 cells. In addition, lnc-PKD2–2-3 was positively correlated with CSC markers in CCA tumor tissues and was markedly upregulated in CCA stem-like cells compared with that in normal CCA cells [140]. Amit K. Srivastava et al. reported that miR-328-3p (miR-328) is significantly upregulated in ovarian CSCs. High expression of miR-328 maintained CSC properties by directly targeting DNA damage binding protein 2 (DDB2), which has been shown previously to inhibit ovarian CSC. Targeting miR-328 could be exploited for the eradication of CSC and aversion of tumor metastasis in ovarian cancer [57].

## Conclusion

The common features of these molecular mechanisms of CSC-mediated therapy resistance are the maintenance of the stemness and dormancy which is the basis for the ability of CSCs to counteract therapy. Other mechanisms include drug efflux mechanism; anti-apoptotic mechanism; DNA damage repair mechanism, and CSC niche, immune evasion by manipulating the TME. By studying the mechanism of resistance, we can explore new targets and improve traditional anti-tumor strategies. The targeted therapy of CSC has greater potential than the traditional therapy to simultaneously eliminate progenitor cells and CSCs, and improve the overall effect of therapy in cancer patients. Current treatment strategies targeting CSCs mainly include specific surface markers and intracellular signal transduction pathways for CSCs, induction of tumor stem cell differentiation, and alteration of TME. The intra/inter tumor heterogeneity and the complexity of the TME make therapy extremely ineffective, therefore, greater understanding of intra/inter tumor heterogeneity and TME is needed for novel therapies to emerge. Future successful eradiation of CSCs and overcome therapy resistance mainly depend on combination therapies that target multiple CSC pathways as well as target proliferating cancer cells.

## Data Availability

Not applicable.

## Abbreviations

GAC: Gastric adenocarcinoma
CSCs: Cancer stem cells
EMT: Epithelial-mesenchymal transition
TME: Tumor micro-environment
ALDH1: Aldehyde dehydrogenase1
HA: Hyaluronic acid
ALCAM: Activated leukocyte cell adhesion molecule
PTC: Papillary thyroid carcinoma
HGF: Hepatocyte growth factor
PTCH: Patched transmembrane receptors
NICD: Notch intracellular domain
CPT1B: Carnitine palmitoyltransferase 1B
FAO: Fatty acid b-oxidation
MDR: Multidrug resistance
MRD: Minimum residual disease
ABC transporter: ATP-binding cassette transporter
ROCK: Rho-associated protein kinase
LncRNAs: Long-chain non-coding RNAs
MSI: Musashi
SGs: Stress granules
TLR4: Toll-like receptors 4
TNBC: Triple-negative breast cancer
CCA: Cholangiocarcinoma
DDB2: Damage binding protein 2
ROS: Reactive oxygen species
RNS: Reactive nitrogen species
ITH: Intratumor heterogeneity
NOD/SCID: Non-obese diabetic /Severe combined immune deficient
